# Genome Analysis and Expression Patterns of Odorant-Binding Proteins from the Southern House Mosquito *Culex pipiens quinquefasciatus*

**DOI:** 10.1371/journal.pone.0006237

**Published:** 2009-07-16

**Authors:** Julien Pelletier, Walter S. Leal

**Affiliations:** Honorary Maeda-Duffey Laboratory, Department of Entomology, University of California Davis, Davis, California, United States of America; University of Maryland, United States of America

## Abstract

Olfactory-based behaviors in mosquitoes are mediated by odorant-binding proteins (OBPs). They form a multigenic family involved in the peripheral events in insect olfaction, specifically the transport of odorants to membrane-bound odorant receptors. OBPs contribute to the remarkable sensitivity of the insect's olfactory system and may be involved in the selective transport of odorants.

We have employed a combination of bioinformatics and molecular approaches to identify and characterize members of the “classic” OBP family in the Southern House mosquito *Culex pipiens quinquefasciatus* ( = *Cx. quinquefasciatus*), a vector of pathogens causing several human diseases. By taking advantage of the recently released genome sequences, we have identified fifty-three putative *Cx. quinquefasciatus* OBP genes by Blast searches. As a first step towards their molecular characterization, expression patterns by RT-PCR revealed thirteen genes that were detected exclusively and abundantly in chemosensory tissues. No clear differences were observed in the transcripts levels of olfactory-specific OBPs between antennae of both sexes using semi-quantitative RT-PCR. Phylogenetic and comparative analysis revealed orthologous of *Cx. quinquefasciatus* OBPs in *Anopheles gambiae* and *Aedes aegypti*. The identification of fifty-three putative OBP genes in *Cx. quinquefasciatus* highlights the diversity of this family. Tissue-specificity study suggests the existence of different functional classes within the mosquito OBP family. Most genes were detected in chemosensory as well as non chemosensory tissues indicating that they might be encapsulins, but not necessarily olfactory proteins. On the other hand, thirteen “true” OBP genes were detected exclusively in olfactory tissues and might be involved specifically in the detection of “key” semiochemicals. Interestingly, in *Cx. quinquefasciatus* olfactory-specific OBPs belong exclusively to four distinct phylogenetic groups which are particularly well conserved among three mosquito species.

## Introduction

In insects, odorants (aka semiochemicals) are detected by specialized sensory structures, the olfactory sensilla, present on different chemosensory tissues such as antennae, maxillary palps and proboscis. Hydrophobic odorant molecules have to pass through an aqueous medium, the sensillar lymph, separating the port of entry on the sensilla (the pore tubules) and receptors neurons. There is now increasing evidence that a multigenic family of small soluble proteins first identified in moths, the odorant-binding proteins (OBPs) [1], is involved in this important process leading to the delivery of odorants to the odorant receptors [2], [3].

A detailed mechanism has been proposed for a pheromone binding protein of the silkmoth, BmorPBP1, suggesting that a pH-dependent conformational change is involved in pheromone binding and release [4], [5], [6], [7]. Indeed, structural biology studies showed that the C-terminal part of the protein forms an additional α-helix at low pH capable to compete with pheromone for the binding pocket [8], [9], [10], thus enabling the delivery of the pheromone in acidic environment similar to that formed by the negatively charged dendrite surfaces of the olfactory receptor neurons [11]. Functional study also showed that BmorPBP1, when co-expressed with pheromone receptor BmorOR1 in the empty neuron system of *Drosophila*, enhanced the response to the pheromone, indicating that OBPs contribute to the inordinate sensitivity of the insect's olfactory system [12].

In mosquitoes, the first OBP (CquiOBP1) was isolated from antennae of female *Culex quinquefasciatus* by native gel electrophoresis and further cloned from cDNA to obtain a full-length sequence [13]. Recently this protein was shown to bind to a mosquito oviposition pheromone [14] in a pH-dependent manner and to be expressed in a subset of sensilla including one type responding to this pheromone [15]. Taken together, these experiments suggest that CquiOBP1 in involved in the detection of semiochemicals involved in mosquito oviposition behavior.

The release of the genome sequences of several insects including three dipteran species has allowed the identification of large multigenic families of OBPs in *Drosophila melanogaster* [16], [17], [18], [19], *Anopheles gambiae* [19], [20], [21], [22] and *Aedes aegypti* [23]. In mosquitoes, different subgroups of OBPs have been identified, each possessing its own characteristic features. The “classic” group includes the majority of OBPs characterized so far and is structurally similar with other insect OBPs. “Classic” OBP genes are predicted to encode small secreted proteins which display a characteristic pattern of six conserved cysteine residues called the “classic motif” [23], as well as a N-terminal signal peptide sequence. Several members of “classic” OBPs have been determined as important components of the insect's chemosensory system, as suggested by their specific association with functionally distinct classes of olfactory sensilla in *D. melanogaster* [24], [25], [26], [27] or by their high expression levels in *A. gambiae* antennae [28], [29]. On the other hand, studies performed on other OBP classes in the malaria mosquito *A. gambiae* revealed that “atypical” OBPs, which possess an extended C-terminal segment, were mostly expressed in early aquatic stages or at very low levels in adult tissues [21], [22], [29], whereas “plus-C” OBPs, which possess at least two additional conserved cysteines, showed no evidence of being olfactory-specific [22], with a few exceptions detected at relatively high levels in antennae [29].

The southern house mosquito *Cx. quinquefasciatus* is an important human health pest as a vector of several pathogens including agents of lymphatic filariasis, West Nile encephalitis and St. Louis encephalitis. In this species only two OBPs have been identified at the molecular level, CquiOBP1 [13] and CquiOBP7 [30], raising the question of how many genes encoding putative OBPs are present. In this study, we have mined the yet to be published genome sequence of *Cx. p. quinquefasciatus* (The genome sequence of *Culex pipiens quinquefasciatus*; *Culex* Genome Consortium), examined the diversity of this multigenic family, and focused on the “classic” OBP genes. Taking advantage of the genomic data, we have identified a total of fifty-three genes encoding putative OBPs in *Cx. quinquefasciatus*. Based on expression studies, we have identified two classes of OBPs, one being specifically expressed in olfactory tissues - and thus suggested to be involved in olfaction (“true” OBPs”) - and an ubiquitous group, encapsulins [2], which might play other physiological role(s).

## Results and Discussion

### Identification of putative “classic” OBP genes

To explore the diversity of the OBP family in the genome of *Cx. quinquefasciatus* (The genome sequence of *Culex pipiens quinquefasciatus*; *Culex* Genome Consortium), we have used the previously identified OBP sequences from other dipteran species (*A. gambiae*, *A. aegypti* and *D. melanogaster*) as probes to look for structurally similar proteins by Blast search [31]. Candidate sequences that displayed significant similarity were manually screened for characteristic features of the OBP family. Several criteria were used to assign a protein sequence as putative OBP: a small size (molecular weight around 14 kDa) and the presence of both a predicted N-terminal signal peptide sequence and highly conserved six cysteines spacing designated as the “classic motif”: C1-X_15-39_-C2-X_3_-C3-X_21-44_-C4-X_7-12_-C5-X_8_-C6 [23], which is now considered as a hallmark of the family. Candidate OBPs were further blasted in NCBI conserved domain database (CDD) to confirm the presence of characteristic motifs conserved in the OBP family.

Homology searches coupled with bioinformatics analysis allowed the identification of fifty-three putative OBP genes in *Cx. quinquefasciatus*, including CquiOBP1 the first ever mosquito OBP characterized [13] and CquiOBP7 recently described as an orthologue of AgamOBP7 [30]. Structural characteristics and GenBank accession numbers of CquiOBP1 to CquiOBP53 are compiled in Table 1. Six proteins had no predicted signal peptide (CquiOBP10, 29, 34, 40, 41, 42), possibly because they lack a full-length N-terminal as suggested by their overall shorter sizes. CquiOBP21 and CquiOBP46 did not fit the “classic motif” of cysteine spacing and CquiOBP45 and CquiOBP47–50 did not match with any conserved OBP domain when blasted in CDD. Yet, these proteins were further analyzed because of their similarity with other mosquito OBPs (see further phylogenetic analysis). CquiOBP45 and CquiOBP50 had been previously identified from salivary glands transcriptome and annotated as “putative salivary odorant-binding proteins” based on their similarity with the C-terminal region of an “atypical” OBP from *A. gambiae* [32]. Both proteins display a slight variation of the “classic motif” as they possess thirteen residues between C4 and C5, a feature they share with five other putative OBPs (CquiOBP44, 47, 48, 49 and 53).

**Table 1:** Structural characteristics of *Cx. quinquefasciatus* putative OBPs.

OBP Name	GenBank accession #	Amino-acids	MW	pI	Cysteine spacing	Signal peptide %	CDD prediction(E-value)
CquiOBP1	AF468212	149/125	14.486	5.52	26/3/37/8/8	98,9	PBP_GOBP (1e-19)
CquiOBP2*	FJ947084	146/124	14.811	5.33	26/3/37/8/8	99,9	PBP_GOBP (4e-23)
CquiOBP3*	FJ947085	147/129	14.539	5.42	27/3/37/8/8/11	95,9	PBP_GOBP (8e-20)
CquiOBP4*	FJ947086	150/132	15.477	5.35	27/27/3/38/8/8	99,9	PBP_GOBP (5e-14)
CquiOBP5*	FJ947087	143/128	14.873	5.01	28/3/38/9/8	87,1	PBP_GOBP (4e-14)
CquiOBP6*	FJ947088	146/125	13.844	8.22	28/3/41/10/8	99,7	PBP_GOBP (5e-17)
CquiOBP7	EU816362	146/126	14.162	5.25	13/12/3/39/8/8/11	1,2	PBP_GOBP (5e-14)
CquiOBP8*	FJ947089	144/121	13.216	8.54	26/3/40/10/8	99,8	PBP_GOBP (1e-12)
CquiOBP9*	FJ947090	147/123	13.826	6.51	28/3/40/10/8	99,9	PBP_GOBP (7e-14)
CquiOBP10	XP_001864761	132	14.734	8.2	26/3/40/10/8	NO	PBP_GOBP (4e-13)
CquiOBP11*	FJ947091	144/121	13.505	8.52	26/3/40/10/8	99,4	PBP_GOBP (5e-17)
CquiOBP12*	FJ947092	146/124	14.364	8.17	17/26/3/40/10/8	92,9	PBP_GOBP (2e-16)
CquiOBP13*	FJ947093	143/120	13.454	5.45	26/3/39/10/8	76,6	PBP_GOBP (1e-16)
CquiOBP14*	FJ947094	170/150	16.797	4.58	45/29/3/33/8/8	100	PhBP (4e-05)
CquiOBP15	XP_001863130	141/113	13.03	4.23	27/3/38/8/8	99,9	PBP_GOBP (6e-08)
CquiOBP16	XP_001863131	134/114	13.043	5.38	27/3/38/8/8	100	PBP_GOBP (5e-08)
CquiOBP17	XP_001863132	132/114	12.577	4.99	27/3/38/8/8	99,9	PBP_GOBP (2e-16)
CquiOBP18	XP_001863133	132/114	12.841	4.92	28/3/38/7/8	100	PBP_GOBP (2e-10)
CquiOBP19	XP_001863134	139/122	13.451	4.76	27/3/38/7/8	100	PBP_GOBP (2e-12)
CquiOBP20	XP_001863135	131/113	12.246	8.5	27/3/38/7/8	100	PBP_GOBP (2e-14)
CquiOBP21	XP_001863136	139/118	13.808	5	31/38/10/5	99,2	PhBP (0,001)
CquiOBP22	XP_001863137	131/112	12.795	4.68	27/3/38/7/8	98,5	PBP_GOBP (1e-11)
CquiOBP23	XP_001843653	136/119	13.3	5.49	29/3/39/8/8	100	PBP_GOBP (7e-08)
CquiOBP24	XP_001864828	137/114	12.957	8.22	28/3/38/7/8	96,6	PBP_GOBP (1e-13)
CquiOBP25	XP_001857294	121/105	12.481	5.59	26/3/41/8/8	99,2	PBP_GOBP (2e-05)
CquiOBP26	XP_001857301	119/104	12.109	4.71	26/3/41/8/8	99,9	PBP_GOBP (4e-08)
CquiOBP27	XP_001857326	126/105	12.042	6.99	26/3/42/8/8	99,7	PBP_GOBP (2e-04)
CquiOBP28	XP_001867251	150/130	14.556	4.5	26/3/42/8/8	100	PBP_GOBP (1e-05)
CquiOBP29	XP_001867252	130	14.624	6.82	26/3/42/8/8	NO	PBP_GOBP (5e-07)
CquiOBP30	XP_001867253	143/123	13.828	5.32	26/3/42/8/8	100	PBP_GOBP (4e-04)
CquiOBP31	XP_001849401	124/108	12.379	4.5	26/3/39/8/8	99,9	PBP_GOBP (2e-08)
CquiOBP32	XP_001866636	126/108	12.096	5.06	26/3/44/8/8	99,5	PBP_GOBP (1e-07)
CquiOBP33	XP_001870016	124/105	12.052	4.5	26/3/42/8/8	99,9	PBP_GOBP (7e-08)
CquiOBP34	XP_001870017	116	12.816	4.94	26/3/39/8/8	NO	PBP_GOBP (2e-05)
CquiOBP35	XP_001870018	126/108	12.039	5.67	26/3/42/8/8	97,2	PBP_GOBP (1e-04)
CquiOBP36	XP_001870019	146/128	13.97	5.01	26/3/42/8/8/7	100	PBP_GOBP (0,003)
CquiOBP37	XP_001849733	135	14.846	8.98	26/3/42/8/8/18	NO	PBP_GOBP (2e-05)
CquiOBP38	XP_001849734	137/117	12.802	4.76	26/3/42/8/8/7	100	PBP_GOBP (0,002)
CquiOBP39	XP_001849735	126/108	12.053	5.9	26/3/42/8/8	98,8	PBP_GOBP (9e-06)
CquiOBP40	XP_001849736	107	11.773	4.69	26/3/39/8/8	NO	PBP_GOBP (9e-05)
CquiOBP41	XP_001849737	98	11.012	6.82	3/41/8/8	NO	PBP_GOBP (8e-07)
CquiOBP42	XP_001849738	111	12.609	5.12	27/3/42/8/8	NO	PBP_GOBP (3e-06)
CquiOBP43	XP_001867883	138/122	14.123	4.86	26/3/38/9/8	100	PBP_GOBP (5e-19)
CquiOBP44	XP_001870734	147/127	14.577	8.73	26/3/41/13/8	91,5	PBP_GOBP (4e-09)
CquiOBP45	AAR18456	139/117	13.209	4.7	26/3/41/13/8	99,8	NO CD (salivary)
CquiOBP46	XP_001861423	150/128	15.071	7.82	26/3/38/20/8	99,2	PBP_GOBP (1e-05)
CquiOBP47	XP_001861424	142/122	14.112	5.51	28/3/38/13/8	100	NO CD (salivary)
CquiOBP48	XP_001861425	139/117	13.153	5.78	26/3/41/13/8	99,9	NO CD (salivary)
CquiOBP49	XP_001861426	143/123	14.094	5.34	26/3/38/13/8	99,9	NO CD (salivary)
CquiOBP50	AAR18408	148/126	14.678	5.23	28/3/38/13/8	99,9	NO CD (salivary)
CquiOBP51	XP_001861428	144/122	13.954	5.33	26/3/38/9/1/8	100	PhBP (1e-04)
CquiOBP52	XP_001861429	143/122	14.359	5.68	26/3/38/10/1/8	100	PBP_GOBP (2e-05)
CquiOBP53	XP_001861430	145/126	14.439	4.83	27/3/36/13/8	98,9	PBP_GOBP (2e-06)

The number of amino acids is indicated for complete/mature proteins. Molecular weights (MW) and isoelectric points (pI) values were predicted for mature proteins using ExPASy server. Cysteine spacing patterns were determined manually. The signal peptides probabilities were predicted using SignalP 3.0 server. Conserved protein motifs result from Blast in NCBI Conserved Domain Database (CDD) with associated E-values. Asterisks indicate when new GenBank accessions have been submitted. Corresponding GenBank accessions: CquiOBP1 (XP_001848926), CquiOBP2 (XP_001848939), CquiOBP3 (XP_001848933), CquiOBP4 (XP_001843595), CquiOBP5 (XP_001848930), CquiOBP6 (XP_001850448), CquiOBP7 (XP_001843143), CquiOBP8 (XP_001851195), CquiOBP9 (XP_001867234), CquiOBP11 (XP_001848048), CquiOBP12 (XP_001867235), CquiOBP13 (XP_001867238), CquiOBP14 (XP_001851213), putative salivary OBP1 AAR18408 (XP_001861427), putative salivary OBP2 AAR18456 (XP_001867923).

**Figure 1:**
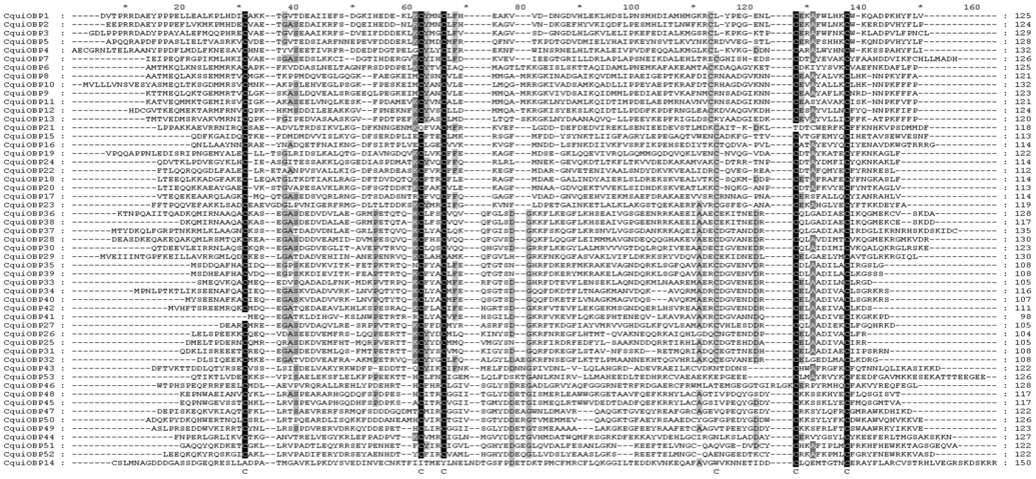
Amino acids alignment of *Cx. quinquefasciatus* putative OBPs. Residues conservation is indicated by different levels of shading: dark grey: 90% conservation; medium grey: 60% conservation; light gray: 40% conservation. The conserved cysteine residues are indicated by the letter C below the alignment. GenBank accession numbers are available in Table 1.

An amino acid alignment of mature *Cx. quinquefasciatus* putative OBPs highlights the very low average identity of this highly divergent multigenic family (Fig. 1). Only the six cysteine residues are fully conserved in each protein, the conservation of C4 being less visible on the alignment because of a more flexible number of residues between C3 and C4 and between C4 and C5.

We have carried out cloning and sequencing of nine genes, CquiOBP3, 4, 5, 8, 9, 11, 12, 13 and 14 to add to four previously characterized OBP genes, CquiOBP1 [13], CquiOBP2 and CquiOBP6 (Ishida and Leal, unpublished data), and CquiOBP7 [30], and two putative salivary odorant-binding proteins CquiOBP45 and CquiOBP50 [32]. The other putative OBPs identified in this study originate from VectorBase automated annotations and were not confirmed by cDNA cloning. Most cloned sequences were similar to VectorBase annotations and only three genes (CquiOBP6, 9, 12) differed from corresponding predicted genes. All new sequences were deposited into GenBank (Table 1).

This bioinformatics-based approach likely gives a good estimation of the range of the OBP family in *Cx. quinquefasciatus*. Multigenic families of “classic” OBPs have now been identified in three different mosquito species with thirty-three genes in *A. gambiae* [20], [21], [22], [23], thirty-four genes in *A. aegypti* [23] and fifty-three genes in *Cx. quinquefasciatus* (this study). This diversity and high divergence of OBP encoding genes in mosquito might be correlated with the structural diversity of semiochemicals perceived by their olfactory system and thus suggest differential affinities for OBPs towards these odorant molecules. Of particular notice, three OBPs that we have already isolated and cloned from *A. aegypti* [33] have been renamed [23]. Thus, previously identified AaegOBP1, 2, and 3 have been renamed AaegOBP39, 27, 56, respectively [34].

### Phylogenetic analysis of mosquito OBPs

**Figure 2:**
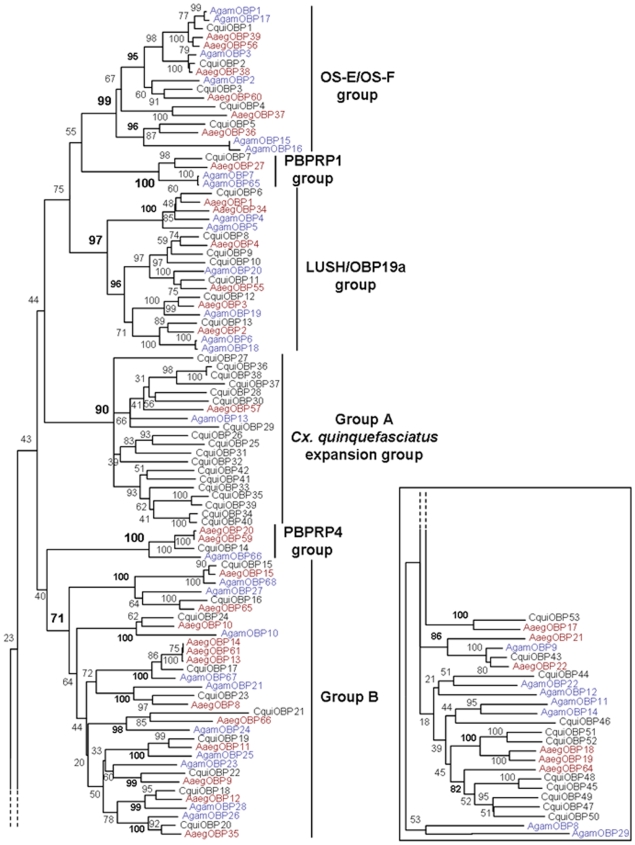
Phylogenetic relationships of mosquito “classic” OBPs. The unrooted consensus tree was generated with 1000 bootstrap replicates using the neighbor joining method. *Cx. quinquefasciatus* OBPs are in black, *A. gambiae* OBPs are in blue and *A. aegypti* OBPs are in red. *A. gambiae* and *A. aegypti* OBPs follow the nomenclature established in [21] and [23]. Robust groupings identified by high bootstrap values at nodes are indicated in bold.

In order to gain insight of the relationships among mosquito OBPs, we have carried out a phylogenetic analysis using putative amino acid sequences. A consensus sequence comparison tree was constructed by the neighbor joining method [35] with one thousand bootstrap replicates. The resulting tree suggests that based on their amino acid identity, most mosquito OBPs are clustered into different groups, each comprising related proteins of the three mosquito species (Fig. 2).

**Figure 3:**
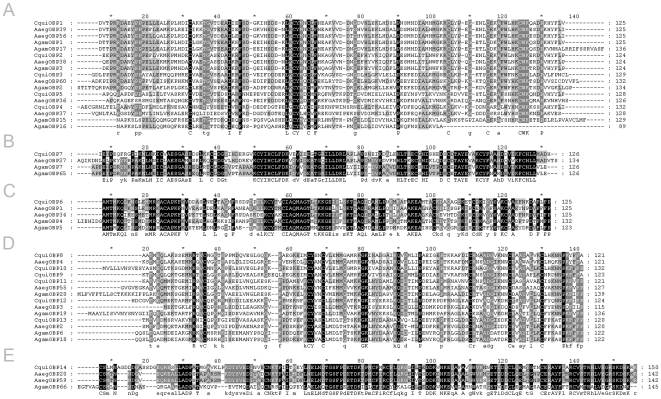
Amino acids alignments of five groups of mosquito OBPs. (A) OS-E/OS-F-like OBPs; (B) PBPRP1-like OBPs; (C) LUSH-like OBPs; (D) OBP19a-like OBPs; (E) PBPRP4-like OBPs. Residues conservation is indicated by different levels of shading: dark grey: 100% conservation; medium gray: 80% conservation; light gray: 60% conservation.

Among these groups, several OBPs of *Cx. quinquefasciatu*s share high identity with other dipterans OBPs already described in previous works, as indicated by the amino acid identity percentages compiled in Table 2. These groups of orthologous proteins have been named OS-E/OS-F, LUSH/OBP19a, PBPRP1, and PBPRP4 based on their similarities to *D. melanogaster* OBPs [20], [21], [22], [23], [36]. In *Cx. quinquefasciatus*, five proteins (CquiOBP1 to CquiOBP5) cluster within the OS-E/OS-F group, one (CquiOBP7) within the PBPRP1 group, one (CquiOBP6) within the LUSH group, six (CquiOBP8 to CquiOBP13) within the OBP19a group, and one (CquiOBP14) within the PBPRP4 group. All these groups are strongly supported by high bootstrap values ranging from 97 to 100%. Amino acid alignments of mosquito OBPs from these groups are provided in Figure 3. Other *Cx. quinquefasciatus* OBPs, mostly in group B, also share high identity with OBPs from other mosquito species (Table 2). Group B is not as strongly supported as others (71% bootstrap support) and encloses nine different subgroups of orthologous OBPs (98 to 100% bootstrap supports). Group A (90% bootstrap support) provides an unexpected example of gene expansion in *Cx. quinquefasciatus*, enclosing eighteen OBPs of this species (CquiOBP25 to CquiOBP42) all related to AgamOBP13 and AaegOBP57. This expansion is a possible explanation for the highest number of putative OBPs identified in *Cx. quinquefasciatus* compared to those found in other mosquito species. The remaining OBPs share less amino acid identity and are not clustered together but rather dispersed at the bottom of the tree. Some of those are classified as putative “salivary” OBPs in NCBI database (Table 1). Among these proteins, CquiOBP53, 52, 51 50, 49 and 47 display some identity with AaegOBP17, 18, 19 and 64 considered so far as *A. aegypti* specific [23], but far less with *A. gambiae* OBPs (Table 2). Overall, *Cx. quinquefasciatus* OBPs are more closely related to *A. aegypti* than *A. gambiae* OBPs, reflecting the fact that both *Culex* and *Aedes* species belong to the same *Culicidae* subfamily.

**Table 2:** Homology relationships of *Cx. quinquefasciatus* with other mosquito OBPs.

OBP Name	Phylogenetic group	*A. gambiae* homolog	Protein identity	*A. aegypti* homolog	Protein identity	*D. melanogaster* homolog	Protein identity
CquiOBP1	OS-E/OS-F	AgamOBP1/17	**90%/79%**	AaegOBP56/39	**88%/87%**	OS-E/OS-F	**64%/64%**
CquiOBP2	OS-E/OS-F	AgamOBP3	**91%**	AaegOBP38	**94%**	OS-E/OS-F	**51%/51%**
CquiOBP3	OS-E/OS-F	AgamOBP2	**53%**	AaegOBP60	**64%**	OS-F	44%
CquiOBP4	OS-E/OS-F	AgamOBP2	39%	AaegOBP37	**53%**	OS-F	31%
CquiOBP5	OS-E/OS-F	AgamOBP3/15	38%/37%	AaegOBP36	**58%**	OS-E	36%
CquiOBP6	LUSH	AgamOBP4/5	**62%/60%**	AaegOBP1/34	**73%/68%**	LUSH	40%
CquiOBP7	PBPRP1	AgamOBP7/65	**55%/54%**	AaegOBP27	**66%**	PBPRP1	28%
CquiOBP8	OBP19a	AgamOBP20	46%	AaegOBP4	**76%**	OBP19a	33%
CquiOBP9	OBP19a	AgamOBP20	42%	AaegOBP4	**67%**	OBP19a	40%
CquiOBP10	OBP19a	AgamOBP20	45%	AaegOBP4	**56%**	OBP19a	34%
CquiOBP11	OBP19a	AgamOBP20	**61%**	AaegOBP55	**70%**	OBP19a	39%
CquiOBP12	OBP19a	AgamOBP19	**60%**	AaegOBP3	**74%**	OBP19a	41%
CquiOBP13	OBP19a	AgamOBP6/18	**62%/62%**	AaegOBP2	**71%**	OBP19a	30%
CquiOBP14	PBPRP4	AgamOBP66	**50%**	AaegOBP20/59	**74%/74%**	PBPRP4	28%
CquiOBP15	B	AgamOBP68	**85%**	AaegOBP15	**92%**		
CquiOBP16	B	AgamOBP27	42%	AaegOBP65	**65%**		
CquiOBP17	B	AgamOBP67	**67%**	AaegOBP13/14	**77%/77%**		
CquiOBP18	B	AgamOBP28	**62%**	AaegOBP12	**76%**		
CquiOBP19	B	AgamOBP25	**53%**	AaegOBP11	**72%**		
CquiOBP20	B	AgamOBP26	**67%**	AaegOBP35	**81%**		
CquiOBP21	B	AgamOBP24	24%	AaegOBP66	37%		
CquiOBP22	B	AgamOBP23	41%	AaegOBP9	**60%**		
CquiOBP23	B	AgamOBP21	42%	AaegOBP8	**61%**		
CquiOBP24	B	AgamOBP10	47%	AaegOBP10	**66%**		
CquiOBP25	A	AgamOBP13	26%	AaegOBP57	21%		
CquiOBP26	A	AgamOBP13	27%	AaegOBP57	22%		
CquiOBP27	A	AgamOBP13	30%	AaegOBP57	31%		
CquiOBP28	A	AgamOBP13	47%	AaegOBP57	49%		
CquiOBP29	A	AgamOBP13	34%	AaegOBP57	31%		
CquiOBP30	A	AgamOBP13	38%	AaegOBP57	42%		
CquiOBP31	A	AgamOBP13	29%	AaegOBP57	31%		
CquiOBP32	A	AgamOBP13	32%	AaegOBP57	30%		
CquiOBP33	A	AgamOBP13	30%	AaegOBP57	28%		
CquiOBP34	A	AgamOBP13	30%	AaegOBP57	26%		
CquiOBP35	A	AgamOBP13	29%	AaegOBP57	29%		
CquiOBP36	A	AgamOBP13	40%	AaegOBP57	43%		
CquiOBP37	A	AgamOBP13	35%	AaegOBP57	39%		
CquiOBP38	A	AgamOBP13	39%	AaegOBP57	43%		
CquiOBP39	A	AgamOBP13	30%	AaegOBP57	28%		
CquiOBP40	A	AgamOBP13	29%	AaegOBP57	27%		
CquiOBP41	A	AgamOBP13	26%	AaegOBP57	24%		
CquiOBP42	A	AgamOBP13	33%	AaegOBP57	34%		
CquiOBP43	-	AgamOBP9	**75%**	AaegOBP22	**77%**	OBP99a	41%
CquiOBP44	-	AgamOBP22	27%	AaegOBP21	23%		
CquiOBP45	-	AgamOBP12	16%	AaegOBP64	25%		
CquiOBP46	-	AgamOBP11	19%	AaegOBP18/19	18%/17%		
CquiOBP47	-	AgamOBP9	22%	AaegOBP64	36%		
CquiOBP48	-	AgamOBP22	17%	AaegOBP64	24%		
CquiOBP49	-	AgamOBP22/14	16%/16%	AaegOBP64	32%		
CquiOBP50	-	AgamOBP12	17%	AaegOBP64	33%		
CquiOBP51	-	AgamOBP9	22%	AaegOBP19/18	36%/35%		
CquiOBP52	-	AgamOBP9	20%	AaegOBP19/18	38%/37%		
CquiOBP53	-	AgamOBP22	22%	AaegOBP17	44%		

Amino acids identity percentages were calculated using GeneDoc software. *A. gambiae* and *A. aegypti* OBPs follow the nomenclature established in [21] and [23]. *Drosophila melanogaster* OBPs displaying at least 25% identity were included: OS-E (DmelOBP83b, NP_524242); OS-F (DmelOBP83a, NP_524241); PBPRP1 (DmelOBP69a, NP_524039); LUSH (DmelOBP76a, NP_524162); OBP19a (DmelOBP19a, NP_728338); PBPRP4 (DmelOBP84a, NP_476990); OBP99a (DmelOBP99a, NP_651707). Amino-acids identities over 50% are in bold. Phylogenetic groups are derived from Figure 2.

Comparative analysis highlights several highly related proteins in *Culex*, *Anopheles* and *Aedes*, as well as other proteins much less conserved among these three species. It is tempting to speculate that highly conserved OBPs should perform a common role within all species. However conservation of sequences does not necessarily imply conservation of functions, and only further functional experiments could shed light on common roles of mosquito highly “homologous” OBPs. Likewise, divergent OBPs will have to be investigated to support their potential implication in species-specific roles.

### Genomic organization of putative OBP genes

Genomic organization was studied according to the relative positions of genes on genomic supercontigs and revealed that most OBP genes (thirty-six of fifty-three) are not distributed randomly in the genome but organized in clusters of genes (Table 3). Eight different clusters ranging from two to eight genes were identified. The most important in term of number of genes are cluster #8 on contig 3.315 regrouping eight genes (CquiOBP46 to CquiOBP53) within 16 kb, cluster #3 on contig 3.424 regrouping eight genes (CquiOBP15 to CquiOBP22) within 69 kb, cluster #5 on contig 3.181 regrouping six genes (CquiOBP37 to CquiOBP42) within 33 kb, and cluster #4 on contig 3.1894 regrouping four genes (CquiOBP33 to CquiOBP36) within 26 kb. Two OS-E/OS-F-like genes (CquiOBP3, 5) are also located at close range on supercontig 3.150 (cluster #1), as well as three OBP19a-like genes (CquiOBP9, 12, 13) on supercontig 3.865 (cluster #2).

**Table 3:** Genomic organization of *Cx. quinquefasciatus* OBP genes.

OBP Name	VectorBase accession #	Supercontig	Genomic position	Cluster #
CquiOBP1	CPIJ007604	3.150	170,719–174,721	-
CquiOBP2	CPIJ007617	3.150	672,931–673,546	-
CquiOBP3	CPIJ007611	3.150	540,281–542,064	1
CquiOBP4	CPIJ001730	3.25	734,060–734,572	-
CquiOBP5	CPIJ007608	3.150	516,885–517,412	1
CquiOBP6*	CPIJ008793	3.206	489,697–490,937	-
CquiOBP7*	CPIJ001365	3.18	1720,262–1721,216	-
CquiOBP8	CPIJ009568	3.240	122,626–123,234	-
CquiOBP9*	CPIJ016948	3.865	41,129–46,297	2
CquiOBP10	CPIJ013976	3.550	256,165–256,681	-
CquiOBP11	CPIJ006551	3.121	270,272–277,928	-
CquiOBP12*	CPIJ016949	3.865	46,518–47,165	2
CquiOBP13	CPIJ016952	3.865	54,944–61,815	2
CquiOBP14	CPIJ009586	3.240	569,948–574,407	-
CquiOBP15	CPIJ012714	3.424	103,588–109,982	3
CquiOBP16	CPIJ012715	3.424	112,183–112,979	3
CquiOBP17	CPIJ012716	3.424	113,896–114,578	3
CquiOBP18	CPIJ012717	3.424	122,946–123,411	3
CquiOBP19	CPIJ012718	3.424	131,078–131,864	3
CquiOBP20	CPIJ012719	3.424	135,879–136,509	3
CquiOBP21	CPIJ012720	3.424	171,439–171,968	3
CquiOBP22	CPIJ012721	3.424	172,603–173,060	3
CquiOBP23	CPIJ001876	3.26	255,589–259,525	-
CquiOBP24	CPIJ014525	3.561	24,869–25,524	-
CquiOBP25	CPIJ010723	3.286	224,289–224,718	7
CquiOBP26	CPIJ010724	3.286	228,005–228,420	7
CquiOBP27	CPIJ010728	3.286	489,935–490,384	-
CquiOBP28	CPIJ016965	3.865	148,161–148,975	6
CquiOBP29	CPIJ016966	3.865	149,508–150,489	6
CquiOBP30	CPIJ016967	3.865	154,625–155,111	6
CquiOBP31	CPIJ008285	3.167	404,302–404732	-
CquiOBP32	CPIJ016479	3.770	2,731–3,167	-
CquiOBP33	CPIJ019607	3.1894	15,149–15,587	4
CquiOBP34	CPIJ019608	3.1894	29,115–29,465	4
CquiOBP35	CPIJ019609	3.1894	31,188–31,622	4
CquiOBP36	CPIJ019610	3.1894	41,408–41,883	4
CquiOBP37	CPIJ007931	3.181	460,064–466,993	5
CquiOBP38	CPIJ007932	3.181	467,058–467,528	5
CquiOBP39	CPIJ007933	3.181	481,658–482,092	5
CquiOBP40	CPIJ007934	3.181	487,383–487,920	5
CquiOBP41	CPIJ007935	3.181	488,157–488,453	5
CquiOBP42	CPIJ007936	3.181	492,753–493,384	5
CquiOBP43	CPIJ017326	3.984	153,967–154,634	-
CquiOBP44	CPIJ009937	3.265	418,539–421,106	-
CquiOBP45*	CPIJ017340	3.991	152,854–153,246	-
CquiOBP46	CPIJ010782	3.315	176,953–177,463	8
CquiOBP47	CPIJ010783	3.315	183,640–184,122	8
CquiOBP48	CPIJ010784	3.315	186,427–186,913	8
CquiOBP49	CPIJ010785	3.315	187,165–187,722	8
CquiOBP50	CPIJ010786	3.315	187,841–188,288	8
CquiOBP51	CPIJ010787	3.315	189,941–190,471	8
CquiOBP52	CPIJ010788	3.315	190,549–191,091	8
CquiOBP53	CPIJ010789	3.315	191,345–193,026	8

Accession numbers and positions of genes on genomic supercontigs are from *Cx. quinquefasciatus* VectorBase genome annotations. The different clusters of genes are indicated by different numbers. Asterisks indicate incorrect VectorBase gene annotations.

OBPs of one cluster always belong to the same phylogenetic group, indicating that they share more identity among them than with other OBPs (Fig. 2) (Table 3). From an evolutionary point of view, close localization and sequence conservation inside a cluster suggests that *Cx. quinquefasciatus* OBP gene family might have evolved by multiple gene duplication events followed by rapid diversifications, as already suggested for *A. gambiae* [21] and *A. aegypti* OBP families [23]. Most clustered adjacent genes are located at close range, but genomic data suggest that such events might also result into long range duplications. For example, two OS-E/OS-F-like genes, CquiOBP1 and CquiOBP2 that share 63% amino acid identity and are located on the same supercontig 3.150 are nevertheless separated by more than 342 kb. Another OS-E/OS-F-like gene, CquiOBP4, is not part of cluster #1 but we have found an almost identical partial OBP gene (XP_001848931, CPIJ007609) located between CquiOBP3 and CquiOBP5 on cluster #1, suggesting that CquiOBP4 might have arisen from duplication of this gene. Additionally, we have also found two triplets of adjacent genes located on two different clusters (clusters #4 and #5) sharing around 90% identity between each pair (CquiOBP34 and CquiOBP40, CquiOBP35 and CquiOBP39, CquiOBP36 and CquiOBP38), indicating that a large duplication event involving three genes might have occurred.

Interestingly, eight clustered OBPs (CquiOBP15 to CquiOBP22, cluster #3) share high identity with related proteins in *A. gambiae* (AgamOBP23 to AgamOBP28) and in *A. aegypti* (AaegOBP11 to AaegOBP15 and AaegOBP65, 66), which are also part of a cluster [21], [23] (Table 2). These data suggest that duplication events likely occurred in a common ancestor before the radiation of the three mosquito species. Detailed comparative genomic analysis is now needed to confirm the orthology relationships among mosquito OBPs, as recently demonstrated for PBPRP1-like genes; CquiOBP7, AgamOBP7, and AaegOBP2 [30]. (Note that the protein referred here as AaegOBP2 [23] is not the previously isolated AaegOBP2 [33], which has been renamed AaegOBP27 [34]).

### Expression patterns in different tissues

Tissue-specificity of forty-seven OBP genes was studied by non-quantitative RT-PCR to determine expression profile of the OBP family members in *Cx. quinquefasciatus*. Expression studies represent an important step to determine if putative OBPs are potentially involved in odorant reception. This assumption is supported by the fact that hitherto all OBPs with identified function have been demonstrated to be expressed only in olfactory tissues. There are a number of OBP-like proteins expressed in non-olfactory tissues, but their olfactory functions have never been demonstrated or even examined [2]. Our assumption is that a gene abundantly and exclusively detected in chemosensory tissues likely encodes an olfactory protein. Gene-specific primers of forty-seven OBPs were used in PCR reactions using cDNA templates prepared from adult antennae, maxillary palps, proboscis, legs and bodies of both sexes. Four genes (CquiOBP34, 40, 41, 42) were not included in the experiment and two pairs of highly similar genes (CquiOBP35/39 and CquiOBP36/38) were considered as single genes. Two distinct cDNA pools were tested, one-day-old and one-to-seven-days old adults. No bands corresponding to genomic DNA amplification were observed, confirming the quality of cDNA samples. In order to examine the transcripts levels between olfactory and non-olfactory tissues, specific primers of a “housekeeping” gene encoding ribosomal protein L8 (CquiRpL8) were used as control to check the integrity of each cDNA preparation.

Non-quantitative RT-PCR experiments showed a high variability in the expression profiles of putative OBP genes, with considerable variations both in tissue distributions and also in term of expression levels. Comparison between sexes did not show a single sex-specific gene, and no differences were observed between one-day-old and one-to-seven-days-old adults. Results are compiled in Table 4 which lists the presence or absence of the expected PCR product for each gene in different tissues.

**Table 4:** Expression patterns of OBP genes by RT-PCR in adult tissues.

OBP Name	Antennae	Maxillary palps	Proboscis	Legs	Bodies	Expression patterns
**CquiOBP1**	Yes	Yes	Yes	No	No	Olfactory-specific
**CquiOBP2**	Yes	No	No	No	No	Olfactory-specific
**CquiOBP3**	Yes	No	No	No	No	Olfactory-specific
**CquiOBP4**	Yes	Yes	Yes	No	No	Olfactory-specific
**CquiOBP5**	Yes	No	No	No	No	Olfactory-specific
**CquiOBP6**	Yes	Yes	Yes	No	No	Olfactory-specific
**CquiOBP7**	Yes	Yes	No	No	No	Olfactory-specific
**CquiOBP8**	Yes	Yes	Yes	No	No	Olfactory-specific
**CquiOBP9**	Yes	No	No	No	No	Olfactory-specific
CquiOBP10	Yes	Yes	Yes	Yes	No	Non olfactory-specific
**CquiOBP11**	Yes	Yes	Yes	No	No	Olfactory-specific
**CquiOBP12**	Yes	No	No	No	No	Olfactory-specific
**CquiOBP13**	Yes	Yes	Yes	No	No	Olfactory-specific
**CquiOBP14**	Yes	No	No	No	No	Olfactory-specific
CquiOBP15	No	No	No	No	No	Not detected
CquiOBP16	No	No	No	No	No	Not detected
CquiOBP17	Yes	No	Yes	Yes	Yes	Non olfactory-specific
CquiOBP18	Yes	Yes	No	Yes	Yes	Non olfactory-specific
CquiOBP19	Yes	Yes	Yes	Yes	Yes	Ubiquitous
CquiOBP20	Yes	Yes	Yes	Yes	Yes	Ubiquitous
CquiOBP21	Yes	Yes	Yes	Yes	Yes	Ubiquitous
CquiOBP22	Yes	No	Yes	Yes	Yes	Non olfactory-specific
CquiOBP23	No	No	No	No	No	Not detected
CquiOBP24	Yes	Yes	Yes	Yes	Yes	Ubiquitous
CquiOBP25	Yes	No	No	Yes	No	Non olfactory-specific
CquiOBP26	Yes	No	No	Yes	No	Non olfactory-specific
CquiOBP27	No	No	No	No	No	Not detected
CquiOBP28	No	Yes	Yes	Yes	No	Non olfactory-specific
CquiOBP29	Yes	Yes	Yes	Yes	Yes	Ubiquitous
CquiOBP30	Yes	Yes	Yes	Yes	Yes	Ubiquitous
CquiOBP31	No	No	No	No	No	Not detected
CquiOBP32	No	No	No	No	No	Not detected
CquiOBP33	No	No	Yes	Yes	No	Non olfactory-specific
CquiOBP34	Not done	Not done	Not done	Not done	Not done	-
CquiOBP35	No	No	No	No	No	Not detected
CquiOBP36	No	No	No	Yes	No	Non olfactory-specific
CquiOBP37	No	No	No	No	No	Not detected
CquiOBP38	No	No	No	Yes	No	Non olfactory-specific
CquiOBP39	No	No	No	No	No	Not detected
CquiOBP40	Not done	Not done	Not done	Not done	Not done	-
CquiOBP41	Not done	Not done	Not done	Not done	Not done	-
CquiOBP42	Not done	Not done	Not done	Not done	Not done	-
CquiOBP43	Yes	Yes	Yes	No	Yes	Non olfactory-specific
CquiOBP44	Yes	Yes	No	Yes	No	Non olfactory-specific
CquiOBP45	No	Yes	No	Yes	Yes	Non olfactory-specific
CquiOBP46	No	Yes	No	Yes	Yes	Non olfactory-specific
CquiOBP47	No	Yes	No	No	Yes	Non olfactory-specific
CquiOBP48	Yes	Yes	Yes	No	Yes	Non olfactory-specific
CquiOBP49	Yes	Yes	Yes	No	Yes	Non olfactory-specific
CquiOBP50	Yes	Yes	Yes	Yes	Yes	Ubiquitous
CquiOBP51	Yes	Yes	Yes	Yes	Yes	Ubiquitous
CquiOBP52	Yes	Yes	Yes	Yes	Yes	Ubiquitous
CquiOBP53	No	No	No	No	No	Not detected
Total number of genes detected	32	26	23	22	18	13 = Olfactory-specific; 16 = Olfactory and non olfactory tissues; 9 = Ubiquitous; 9 = Not detected

Specific primers of forty-seven putative OBP genes were used in non quantitative RT-PCR experiments using thirty-four cycles of amplification. Yes: a PCR product of the expected size has been detected in a given tissue; No: absence of band. The same primer pairs have been used for CquiOBP35 and CquiOBP39, and for CquiOBP36 and CquiOBP38. Expression patterns are as follows. Olfactory-specific: detected only in antennae, palps or proboscis; non olfactory-specific: detected in antennae, palps or proboscis as well as in legs and/or bodies; ubiquitous: detected in every tissue; not detected. Olfactory-specific OBPs are in bold.

Distribution of *Cx. quinquefasciatus* OBP transcripts highlights heterogeneous expression profiles in olfactory as well as non-olfactory tissues. Thirty-two genes were consistently detected in antennae (68%), twenty-six in maxillary palps (55%) and twenty-three in proboscis (49%) but also twenty-two in legs (47%) and eighteen in bodies (38%). The high proportion of genes detected in the main olfactory organ, the antennae, is consistent with the presence of multiple functional classes of sensilla recently described in *Cx. quinquefasciatus* [37]. Contrary to antennae, maxillary palps harbor a single type of olfactory sensillum that has been shown to respond to a broad spectrum of odorants in *Cx. quinquefasciatus* [38]. Even if co-expression of several OBPs can occur in the same sensillum type [25], [27], the unexpected high number of genes detected in this organ remains to be elucidated. A similar proportion (thirteen of twenty-five genes, 52%) of OBPs was detected in *A. gambiae* maxillary palps by RT-PCR [29]. Proboscis, the main gustatory organ in mosquito, was demonstrated to be an accessory olfactory organ in *A. gambiae*, which expresses at least twenty-four odorant receptor genes and responds to a small set of volatile compounds [39]. Consequently, it is reasonable to assume that such olfactory function might also exist in *Cx. quinquefasciatus* proboscis thus requiring the presence of the diverse group of OBPs observed in this study. Alternatively, OBPs expressed in proboscis may be involved in gustatory reception.

**Figure 4:**
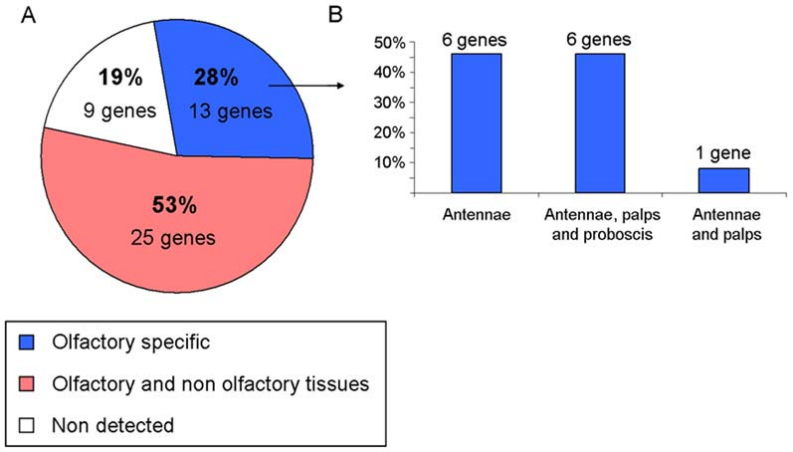
Expression patterns of OBP genes in various tissues of adults *Cx. Quinquefasciatus*. Specific primers of forty-seven putative OBP genes have been used in non quantitative RT-PCR experiments using thirty-four cycles of amplification. (A) OBP genes can be subdivided into three main categories. Olfactory-specific genes were detected exclusively in antennae, maxillary palps or proboscis. (B) Distribution profiles of olfactory-specific genes in olfactory tissues. Details are available in Table 4.

We have classified *Cx. quinquefasciatus* OBPs into different categories according to their expression patterns (Fig. 4). For simplicity, we grouped antennae, maxillary palps and proboscis as olfactory tissues, whereas legs and bodies were considered as non-olfactory tissues. Only thirteen genes (28%) were detected exclusively in olfactory tissues, whereas twenty-five (53%) were detected in olfactory as well as non-olfactory tissues, and nine (19%) were not detected at all. These genes which have not been detected in any adult tissues might represent pseudogenes, may be expressed in earlier stages (which are not the focus of this study), or could be expressed in adults at so low levels that were not detected under the conditions employed in this study. With four independent replications, non-quantitative RT-PCR sufficed to clearly demonstrate differences in bands intensities showing that the most abundant transcripts detected in antennae, maxillary palps and proboscis, belong mainly to the olfactory-specific gene class (data not shown). Among those, CquiOBP1 displayed the highest transcript level in antennae, which is consistent with a previous study showing that CquiOBP1 was the most abundant protein detected in female antennae extracts on a native gel [13]. Based on their high expression levels restricted to chemosensory tissues, we suggest that these thirteen olfactory-specific genes in *Cx. quinquefasciatus* are “true” OBPs, which may be involved specifically in the reception of important olfactory cues.

Among the twenty-five genes detected in both olfactory and non-olfactory tissues, some transcripts were detected at very high levels in legs and/or in bodies indicating that the encoded proteins probably perform some important but non-olfactory functions in these tissues. Interestingly, CquiOBP29 was detected in every tissue but at very high levels in antennae, maxillary palps and proboscis, comparable with some olfactory-specific OBPs. Without any functional evidence, we cannot exclude that genes expressed in olfactory tissues but also in legs and/or in bodies are involved in olfaction, but it is reasonable to consider that proteins involved in the sensitivity and selectivity of the insect's olfactory system are restricted to the sensillar lymph. Some OBPs have been shown to be expressed in broad areas including regions without chemosensory functions, for example in *D. melanogaster* [16] and *A. gambiae* [22], [29]. In *A. aegypti*, AaegOBP22 (close to CquiOBP43 and AgamOBP9) has recently been proposed as a “multi-functions” protein performing different roles in distinct tissues, including non-olfactory functions as suggested by its expression in male reproductive apparatus and in spiracles [40], which are part of the insect's respiratory system. We suggest that this class of broadly expressed OBPs in *Cx. quinquefasciatus* might be encapsulins [2], probably involved in other physiological functions most likely unrelated to odorant reception. On the other hand, the roles of “true” OBPs might be restricted to transport, protection, and delivery of odorants. Test of these hypotheses must await functional studies.

### Correlation between expression patterns and phylogeny

Comparison between expression and phylogenetic data could lead to a better understanding of the role(s) of OBP family in mosquitoes. In *Cx. quinquefasciatus*, olfactory-specific genes (CquiOBP1 to 9, CquiOBP11 to 14) are not distributed randomly in the tree, but along with other mosquitoes related OBPs, belong exclusively to four strongly supported phylogenetic groups: OS-E/OS-F, LUSH/OBP19a, PBPRP1 and PBPRP4 (Fig. 2) (Table 2). These groups, with the exception of one member, CquiOBP10 (an OBP19a-like, which is also detected in legs), constitute groups of exclusively olfactory-specific OBPs in *Cx. quinquefasciatus*. Orthologous proteins in *D. melanogaster* were also shown to be exclusively expressed in chemosensory tissues [16]. In order to study this correlation in another mosquito species and in the absence of expression data for *A. aegypti* OBPs, we have compared our data with other expression studies performed on *A. gambiae* OBPs. Interestingly, all but one of the eleven OBPs characterized in [22] as the most likely to play a role in olfaction (AgamOBP1, 2, 3, 4, 7, 15, 18, 19, 20, 66) belong to the same groups. This comparison was done by semi-quantitative RT-PCR to determine expression levels of *A. gambiae* OBPs in heads, legs and bodies. Results showed that these eleven genes were expressed exclusively or mainly in head tissues. In another study [28], *A. gambiae* antennal cDNA libraries have been characterized by filter array hybridization. Seven OBPs (AgamOBP1, 2, 3, 4, 5, 6, 7) were shown to be the most abundant transcripts in antennal cDNA populations. Additionally, RT-PCR experiment revealed that these genes were exclusively expressed in heads but not in bodies without heads. These OBPs belong also to the same groups (AgamOBP66, the PBPRP4-like was not tested in this study). In a third study [29], the expression patterns and relative abundances of twenty-five “classic” *A. gambiae* OBP genes have been characterized using microarray hybridization, non-quantitative and quantitative RT-PCR. Results notably showed that eight genes (AgamOBP1, 2, 3, 4, 5, 7, 17, 20) belonging to the same groups were among the ten most expressed OBPs in female antennae (AgamOBP66, the PBPRP4-like was not tested in this study). Expression studies are not yet available for *A. aegypti* OBPs.

This comparison suggests the existence of four distinct groups of “true” OBPs in mosquitoes which consistently display high and/or exclusive expression in chemosensory tissues, both in *Cx. quinquefasciatus* (this study) and *A. gambiae*. OBPs from these groups are, therefore, potentially involved in peripheral reception of “key” semiochemicals for mosquito behaviors. Further experiments are now needed to establish their precise localization in chemosensory tissues, to determine in which functional sensilla types they are expressed, and especially to understand which role they play in the olfactory behavior of mosquitoes. Characterization of their binding to relevant ligands and unveiling their structural features may open the door for the identification of novel attractant and/or repellent compounds. Previously, CquiOBP1 (an OS-E/OS-F-like protein) was demonstrated to be an olfactory protein and subsequently used as a molecular target to identify an oviposition attractant, which was then tested in field tests and is currently employed as lure for trapping gravid female mosquitoes [15].

### Comparison of OBPs expression levels between female and male antennae

Non-quantitative RT-PCR screening allowed the identification of thirteen olfactory-specific OBP genes in *Cx. quinquefasciatus* (CquiOBP1 to 9 and CquiOBP11 to 14). To identify which of these genes are more likely involved in sex-specific behavior, we have carried out semi-quantitative RT-PCR experiments and determined more accurately the expression ratios between antennae of both sexes. For such comparison, the choice of a suitable control gene is of paramount importance. We have decided to use two different alternatives, an ubiquitous ribosomal protein encoding gene (CquiRpL8) and the atypical odorant receptor 7 gene (CquiOR7) [41] to normalize the expression levels of antennal cDNA samples. After normalization, specific primers for each OBP and for both control genes were used in standardized PCR reactions. Quantifications of PCR products intensities (reflecting the transcripts levels) were used to calculate the female antennae/male antennae (FA/MA) expression ratio for each OBP as well as for both control genes.

**Figure 5:**
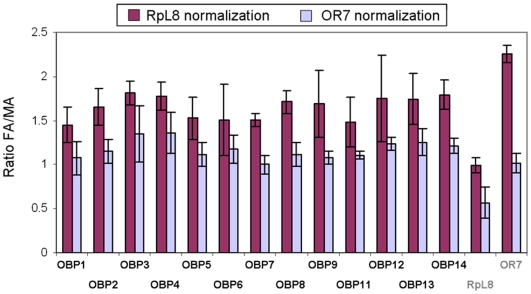
Expression of OBP genes in female and male antennae. Expression ratios (FA/MA) of thirteen olfactory-specific OBP genes and two control genes (RpL8, OR7) were calculated after quantification of bands intensities in semi-quantitative RT-PCR experiments. Antennal CDNAs of both sexes were normalized to the expression levels of CquiRpL8 (purple) and CquiOR7 (blue). Bars represent standard deviations.

**Figure 6:**
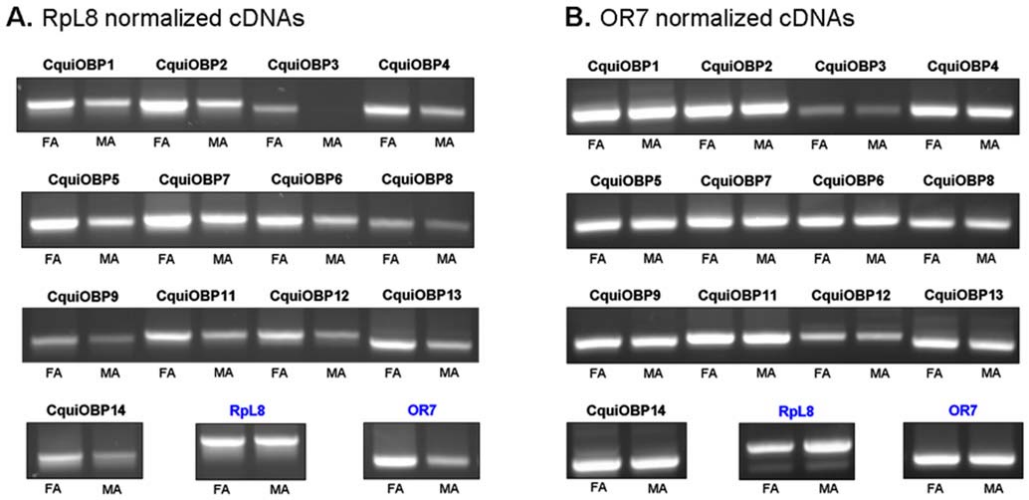
PCR amplification in female and male antennae. Amplification of thirteen olfactory-specific OBP genes and two control genes (RpL8, OR7) in female antennae (FA) and male antennae (MA) cDNAs. (A) cDNAs normalized to the expression levels of CquiRpL8; (B) cDNAs normalized to the expression levels of CquiOR7.

Semi-quantitative RT-PCR data revealed clear differences in OBPs expression ratios in RpL8 compared to OR7 normalized cDNAs (Fig. 5). FA/MA ratios were consistently higher when RpL8 was used as control (OBPs ratios from 1.45 to 1.81, average 1.65) than when OR7 was used as control (OBPs ratios from 1.07 to 1.35, average 1.17). These values likely reflect the difference in the antennal structures in male and female adults. Indeed, in *Culex* mosquitoes, female antennae harbor about three and a half times more olfactory sensilla than male antennae, which harbor sensilla only on the two last distal segments [42]. Thus, the average higher FA/MA value for OBPs in RpL8 normalized cDNAs (1.65) compared to OR7 normalized cDNAs (1.17) might represent an artifact due to a much lower level of OR7 transcript in corresponding male sample. This discrepancy becomes obvious when looking at the transcripts levels of RpL8 and OR7 between sexes. In RpL8 normalized cDNAs, the average FA/MA ratio of OR7 was 2.25, indicating a clear enrichment of OR7 transcript in females. Similarly, in OR7 normalized cDNAs, the average FA/MA ratio of RpL8 was 0.565, indicating a clear enrichment of RpL8 transcript in males. This difference is highlighted in Figure 6 which compares the PCR amplification products of OBPs and control genes in both RpL8 (Fig. 6A) and OR7 (Fig. 6B) normalized cDNAs on agarose gels.

Whereas the “housekeeping” RpL8 gene represents basically per-cell transcripts comparison, OR7 gene might represent a more suitable control to quantify olfactory-specific transcripts ratios considering the structure of *Cx. quinquefasciatus* antennae. This atypical receptor, orthologue of *D. melanogaster* OR83b, is co-expressed with conventional odorant receptors in almost every sensilla type, with the exception of basiconica (grooved pegs) sensilla [41], [43], [44], [45]. Thus, equivalent levels of OR7 transcripts in male and female antennae cDNAs might reflect more accurately equivalent levels of sensilla-specific transcripts, if we assume that both sexes do express the same amount of OR7 transcript in their respective sensilla, which has never been determined in this mosquito species. In *A. gambiae*, a mosquito species which display a similar discrepancy in the number of sensilla between male and female antennae, OR7 has been shown to be expressed about twelve times more in female antennae than in male antennae by quantitative RT-PCR, after normalization by a ribosomal protein (RpS7) [46]. As one would expect about three times higher expressions in female antennae for equally expressed olfactory genes (due to difference in antennal structures), the authors have suggested that a greater proportion of sensilla on female than male antennae might express OR7.

Based only on OR7 normalization, our data show that transcripts levels of olfactory-specific OBPs in *Cx. quinquefasciatus* are relatively similar between antennae of both sexes (OBPs ratios between 1.07 and 1.35) suggesting that none of these genes might be involved directly in sex-specific olfactory behavior in this mosquito species. In *A. gambiae*, mRNA levels of twenty “classic” OBPs have been compared in antennae (or heads) of male and female by microarray hybridization and quantitative RT-PCR after normalization by a ribosomal protein (RpS7), and several transcripts displayed significant enrichment in one or the other sex [29]. It is not clear whether this difference is due to real species-specific variation in OBP expression between *Culex* and *Anopheles*, or to the different control genes used (ribosomal protein VS OR7), or because only a relatively small set of genes (thirteen of thirty-two genes detected in antennae) was tested in our study.

## Materials and Methods

### Identification of putative OBP sequences in *Culex quinquefasciatus*

Predicted peptide sequences database (CpipJ1.2 geneset) of the whole genome of *Cx. quinquefasciatus* (The genome sequence of *Culex pipiens quinquefasciatus*; *Culex* Genome Consortium) was downloaded from VectorBase (http://cpipiens.vectorbase.org/index.php) and entered into BioEdit v7.0.9.0 [47] to perform homology searches using Blastp algorithm [31]. *A. gambiae* (thirty-five sequences), *A. aegypti* (thirty-four sequences) and *D. melanogaster* (thirty-five sequences) “classic” OBP amino-acid sequences were retrieved from GenBank (NCBI) and used as queries in Blast searches. Conservation of the six cysteines spacing pattern and sequence identities with other dipterans OBPs were assessed from multiple alignments using GeneDoc software (http://www.nrbsc.org/gfx/genedoc/ebinet.htm) and BioEdit. N-terminal signal peptide sequences were predicted using SignalP v3.0 server (http://www.cbs.dtu.dk/services/SignalP) [48]. Molecular weights and isoelectric points were computed using ExPASy proteomics server (http://www.expasy.ch/tools/pi_tool.html). Blast in NCBI conserved domains database (CDD) was used to identify PBP_GOBP (pfam01395) or PhBP (smart00708) motifs. Relative positions of putative OBP genes on genomic supercontigs were studied following VectorBase genome annotations. *Cx quinquefasciatus* OBP names (CquiOBP1 to CquiOBP53) were assigned, when possible, based on their phylogenetic relationships and positions on genomic clusters.

### Phylogenetic analysis of mosquito OBPs

Amino acid sequences of putative “classic” OBPs identified in three mosquito species (fifty-three in *Cx. quinquefasciatus* (this study), thirty-three in *A. gambiae* and thirty-four in *A. aegypti*) were used to create an entry file for phylogenetic analysis in MEGA 4.0.2 [49]. An unrooted consensus neighbor joining tree [35] was calculated at default settings with pairwise gaps deletions. Branch support was assessed by bootstrap analysis based on 1000 replicates. Nomenclature of *A. gambiae* and *A. aegypti* OBPs used in phylogenetic analysis was the same as described in [21] and [23].

### Determination of expression patterns by non-quantitative RT-PCR

*Cx. quinquefasciatus* mosquitoes used in this study were from a laboratory colony originating from adult mosquitoes collected in Merced, CA in the 1950s and maintained under laboratory conditions at the Kearney Agricultural Center, University of California, as previously described [38]. Tissues (antennae, maxillary palps, proboscis, legs and bodies) from adults of both sexes were dissected on ice under a light microscope. Total RNA was extracted using TRIzol Reagent (Invitrogen, Carlsbad, CA) and first-strand cDNAs were synthesized from 0.5 µg RNA using SuperScript II Reverse Transcriptase (Invitrogen) and an oligo (dT) primer, following manufacturer's instructions. Integrity of each cDNA template was confirmed by amplification of a “housekeeping” gene encoding ribosomal protein L8 (CquiRpL8, GenBank accession XP_001841927). Gene-specific primers for forty-seven putative *Cx. quinquefasciatus* OBPs were designed manually according to three criteria: spanning at least one predicted intron in order to be able to distinguish between genomic DNA and cDNA amplifications, an annealing temperature around 60°C in order to prevent non-specific amplifications and an expected size around 250–350 bp. PCR reactions were carried out in a GeneAmp PCR System 9700 (Applied Biosystems, Carlsbad, CA) using equivalent amount of cDNA and one unit of Titanium Taq DNA polymerase (Clontech, Palo Alto, CA) in a final volume of 25 µl. After thirty-four cycles of amplification (95°C for 30s, 56°C for 30s, 72°C for 30s), PCR products were loaded onto ethidium-bromide stained agarose gels (1,5% (w/v)) and visualized using a Gel DOC XR Molecular Imager (BioRad, Hercules, CA). Two replicates were performed on two different cDNA samples, one-day-old and one-to-seven-days-old adults. All primers used in RT-PCR experiments are listed in Table 5.

**Table 5:** List of gene specific primers used in RT-PCR experiments.

OBP Name	Primer Forward 5′-3′	Primer Reverse 5′-3′
CquiOBP1	AATTGCTGTTGTTGTGTTGGCGG	GCCAGAATGCTTTCTCGCATAGA
CquiOBP2	CTCATCAGCTGTGAGGAACCGAG	CTTGTTCAGCCAGAATGCCTTCTC
CquiOBP3	ACTTGATGTTCACGCTGGCTGGA	AGGCATCTGCTTCCCATCTTCAG
CquiOBP4	TCTGACGGAGCTTCGAGCGGCTA	GCACGGGCGCAGTTATCATCTCC
CquiOBP5	CCACCAGCCTCGCTAATTGAACT	CATTTGTGGTGAGAAAAGGCTCG
CquiOBP6	CAGTGATGGAGCGATGACGATGA	CGCAAGTTTCCTTGTATCCAGCCT
CquiOBP7	CCGATCAAGATGCTGCACAAGAT	CAGAACTTGATGACATCGTCGTGG
CquiOBP8	ACCATGGAGCAGTTGGCGAAATC	CGCAGCTTCACAGCTGTTCTTCA
CquiOBP9	ACGACCATGGAGCAGTTGCAGAA	CAGAAAGGCATACGCAGCTTCACA
CquiOBP10	GGCGACATGATGCGATCAGTTTGC	CACAGTTGTTCTTGACCCCGTCGC
CquiOBP11	ACCGGCAAAGTTGAGGGTAAAGC	TACACTTGGCCACCGCGTAAGAC
CquiOBP12	TACGCCAAGTTCTGCGGACATGA	CAGTTCAACAGGACGTACGCCGA
CquiOBP13	GACCGTTGAAGACATGAGCCGAG	CAGGTCAACAGCACGTAGGCAAC
CquiOBP14	TGAATGCCGGTGACGACGACGGT	ACCCTCCACCAGATGGCGCGTGC
CquiOBP15	TGGCCGTGCTGATACGACCTAGC	AGAAACGGCCGTCTCGTGGATAC
CquiOBP16	CTTGCTGGCGGCCTACAACAATTG	GTGCCCCACTTGTCTACGGCGTTC
CquiOBP17	GTCACCGAGCAGGAGAAGGAAGC	ATGTAGCACTGCAGCAGGGCAAA
CquiOBP18	CCTGACCGAGGAGCAGCTCAAGA	GTCGTCCATCTTCTGGCTGCACT
CquiOBP19	CCACCCAACCTGGAGGACATCAG	TATACGCCGTATCGCACGCATCC
CquiOBP20	TGACCATCGAGCAGCAGAAGAAG	ACAAGTCCGGCCTTGGTGTTGTA
CquiOBP21	AAGAAGGCCGAAGTCCGGCGGAA	CCGACGGCACCTTGTGGTTCTTGA
CquiOBP22	CAGCGCCAACAAGGGGACCTCTT	ACCTGGCACCGGTCGATCAGAGC
CquiOBP23	ACTCATGCTTTCTTCACCCCGCA	GAAGTAGCACTCGTACAGCCCGTG
CquiOBP24	GATGTGACCAAACTTCCCGACGT	AACATATCGTAGGCCGTGTCGCA
CquiOBP25	CGCAGTCGTGACAGCTGATATGGA	CACACGCGTCGTCATGTTCCGTT
CquiOBP26	GTGCCAAGAGCAGGTGGATGCCT	CCGAAAAAGGCACGCCACAATGT
CquiOBP27	TACTGTACCGTTGGATTGCTGGCA	TTCCGATGCTGCCCAAACAGACA
CquiOBP28	TTCAGGCCGATGAGGCTTCAGAC	CATGCCCTGTTTGACGCAGGTCA
CquiOBP29	TCCTTCTTGCGGTAAGACGTGGC	GCTCACAAAGATCCTCGTTGTCG
CquiOBP30	TTGCGCAGACAGACGAGGAGGTG	TCTGCAGTGCCTGTTTGACGCAG
CquiOBP31	AACTTGTCGCGAACAGGAGGGTG	CAATATCGGCAGCAAGCTCGCAG
CquiOBP32	AAGTGCATGAAAGAGGAGGGCGC	CCCAGCTCACAGCGGTCCTCGTT
CquiOBP33	GTGAAGCAAGCTTGCATGGAACA	AACTCGCAGCGATCCTCGTTCTC
CquiOBP34	Not done	Not done
CquiOBP35*	TTCCATGCCTGCATCGATCAGGA	GCTGCCAGGTCGCAACGGTCTAC
CquiOBP36**	GATTGGACTCTTGCTGGTCTTGGC	AGCTGGCACCGGTCCTCGTTGGT
CquiOBP37	AACTGCAAGTCCAGCGAGGGAGC	CAGCTGGCACCGATCGTCGTTC
CquiOBP38**	GATTGGACTCTTGCTGGTCTTGGC	AGCTGGCACCGGTCCTCGTTGGT
CquiOBP39*	TTCCATGCCTGCATCGATCAGGA	GCTGCCAGGTCGCAACGGTCTAC
CquiOBP40	Not done	Not done
CquiOBP41	Not done	Not done
CquiOBP42	Not done	Not done
CquiOBP43	CTTTACCGTGAAGACCACGGACG	GCAGGTTGTTGGTCTGGAAGCAC
CquiOBP44	CGGTCGTCTGATCAAGGTTTGCA	GATCCGTAAACGCGCTCACAATAC
CquiOBP45	GAGCAACCAAATTGGGGAGAAGT	CTCTTCTTGCAGTAATCGTCTCCG
CquiOBP46	AAGCTCCGCCTGGACCCCGCACT	CGGTAAGGCCGTTCGCACTTCCCC
CquiOBP47	TCGCAGACGAGCCAATCTCCAAG	TCGCAGACGAGCCAATCTCCAAG
CquiOBP48	CGCTACCTCCAAGGAACCAAACT	GTAATGCTTGGAGCTCTTCTTGCA
CquiOBP49	CTATCATTTCCCTCGCCCTGGGA	CTTCTTGCAGTAGTCGTCGCCGT
CquiOBP50	GCGGACCAGAAACCATACGACAA	GCCTTCCAGTCGCACTTGAAGTAC
CquiOBP51	GCGCTCAGCAGTACCAAAAGGAC	GGTATGAACGCCTTGTGGCAGTAA
CquiOBP52	GAACAAAAGCAAAAGTACCGCCAG	AGTACCGCCCAAAACACAGCATC
CquiOBP53	CTATTGGTTCTTGTCGCTGCGGT	ACTCCTTCTTCATCACCGCACCA
CquiRpL8	AGTCGTGAAGCACATCATCCACG	GCCTTACCGATGTGCTGATGGTT
CquiOR7	TCGTCATGGTCATGACGACGACG	CGAAGAGCAGCCAGGAGCAGAAC

Asterisks indicate when the same primers pairs have been used to amplify two different genes with highly similar sequences. Genes encoding ribosomal protein L8 (CquiRpL8, XP_001841927) and odorant receptor 7 (CquiOR7, ABB29301) have been used as controls in non-quantitative RT-PCR (RpL8) and semi-quantitative RT-PCR experiments (RpL8 and OR7).

### Comparison of OBPs expression levels in male and female antennae by semi-quantitative RT-PCR

To compare transcripts levels between antennae of both sexes, antennal cDNA samples (same preparation as described above) were normalized to the expression levels of two different control genes, RpL8 (CquiRpL8, GenBank accession XP_001841927) and OR7 (CquiOR7, GenBank accession ABB29301) [41]. Gradual dilutions and cycle-controlled PCR reactions were used until amplifying equivalent amounts of RpL8 and OR7 in corresponding samples of both sexes. RpL8 and OR7 normalized cDNAs were used in standardized PCR reactions (25 µl, with one unit of Titanium Taq DNA polymerase) with gene-specific primers for thirteen olfactory-specific OBP and for both control genes. All reactions were carried out in the linear range of PCR amplification, as determined for each gene, to prevent saturation bias. PCR products (15 µl) were loaded onto ethidium-bromide stained agarose gels (1.5% (w/v)) and visualized using Gel DOC XR Molecular Imager (BioRad). Quantification of bands intensities was done using Quantity One software (BioRad). Intensity value of each OBP band was divided by those of corresponding control band prepared from the same reaction mix, after background removal. Resulting values were used to calculate the expression ratios between female and male antennae (FA/MA). Three replicates were performed on two different cDNA samples (one-to-seven-days-old adults) for both RpL8 and OR7 normalized samples.

### Cloning and sequencing

Full-length sequences of CquiOBP2 and CquiOBP6 were amplified from female antennal cDNA using Smart Race cDNA amplification kit (Clontech) with specific primers designed from *Culex pipiens* OBP2 and OBP6 genes (unpublished) and universal primers, according to the manufacturer's instructions. Full-length sequences of nine putative OBP genes (CquiOBP3, 4, 5, 8, 9, 11, 12, 13, 14) were amplified from female antennal cDNA using Pfu Ultra II polymerase (Stratagene, La Jolla, CA) with specific primers designed in 5′ and 3′ ends of predicted genes (see below). PCR products were gel purified using QIAquick Gel Extraction Kit (Qiagen, Valencia, CA) and ligated into pBluescript SK (Stratagene). Ligation products were used to transform One Shot OmniMAX competent cells (Invitrogen) and positive clones were grown in LB medium containing ampicilline. Plasmids were purified using QIAprep Spin Miniprep Kit (Qiagen) and sent to Davis Sequencing Inc (Davis, CA). Sequences of all these genes were deposited into GenBank. Accession numbers are available in Table 1.

3′-RACE-CquiOBP2: 5′-GGCCGGCGTGGTGAACGACAAGGGCG-3′
5′-RACE-CquiOBP2: 5′-GCCTTCTCGCACAGATTCTCGCCCTGTGGG-3′
3′-RACE-CquiOBP6: 5′-CCGATCCGATCCCGACCCCGAACTC-3′
5′-RACE-CquiOBP6: 5′-GAGTTCGGGGTCGGGATCGGATCGG-3′
fl-CquiOBP3 forward: 5′-ATGATCATACTCAGTATGGGGTTGCTA-3′
fl-CquiOBP3 reverse: 5′-CTATAGGCAATTTGGAAAGAGCACT-3′
fl-CquiOBP4 forward: 5′-ATGTCGTACAAGTTGCTTGTGCTAGCT-3′
fl-CquiOBP4 reverse: 5′-TCAAATGAGAAAGTAATGAGCTGGA-3′
fl-CquiOBP5 forward: 5′-ATGACGGTGGCCACCTGGTTATCT-3′
fl-CquiOBP5 reverse: 5′-TCAAAACAGGTAATAGTGGACCGG-3′
fl-CquiOBP8 forward: 5′-ATGATCTGGCGAAGGTTTGCGATT-3′
fl-CquiOBP8 reverse: 5′-TTAAGCGAAGAAATATTTGGGGTTAT-3′
fl-CquiOBP9 forward: 5′-ATGAGTGTTCGCGCATTTCTTCCG-3′
fl-CquiOBP9 reverse: 5′-TTACGCAAAGAAAAACTTGGGATTA-3′
fl-CquiOBP11 forward: 5′-ATGGCCACTCGGGTGGAGCTGGCT-3′
fl-CquiOBP11 reverse: 5′-CTAGGGAAACACAAACTTGGGGTTG-3′
fl-CquiOBP12 forward: 5′-ATGAAGTGCGACAGTTGGGCCACC-3′
fl-CquiOBP12 reverse: 5′-CTAGGGGAAAATAAACTTTGGATTGT-3′
fl-CquiOBP13 forward: 5′-ATGCGATATCTAGTGATTTTAGCCATCG-3′
fl-CquiOBP13 reverse: 5′-CTACGGGAAAAAGAACTTGGGCGT-3′
fl-CquiOBP14 forward: 5′-ATGGGTGTCAAAACGGTGATCTTC-3′
fl-CquiOBP14 reverse: 5′-TTATCGCCTTTTGCTGTCCTTGCT-3′
